# Myelosuppression Alleviation and Hematopoietic Regeneration by Tetrahedral‐Framework Nucleic‐Acid Nanostructures Functionalized with Osteogenic Growth Peptide

**DOI:** 10.1002/advs.202202058

**Published:** 2022-07-26

**Authors:** Tianxu Zhang, Mi Zhou, Dexuan Xiao, Zhiqiang Liu, Yueying Jiang, Maogeng Feng, Yunfeng Lin, Xiaoxiao Cai

**Affiliations:** ^1^ State Key Laboratory of Oral Diseases National Clinical Research Center for Oral Diseases West China Hospital of Stomatology Sichuan University Chengdu 610041 P. R. China; ^2^ Department of Oral and Maxillofacial Surgery The Affiliated Stomatology Hospital of Southwest Medical University Luzhou 646000 P. R. China

**Keywords:** bone marrow stromal cells, hematopoietic regeneration, myelosuppression, osteogenic growth peptides, tetrahedral framework nucleic acids

## Abstract

As major complications of chemoradiotherapy, myelosuppression and hematopoietic‐system damage severely affect immunologic function and can delay or even terminate treatment for cancer patients. Although several specific cytokines have been used for hematopoiesis recovery, their effect is limited, and they may increase the risk of tumor recurrence. In this study, osteogenic growth peptide functionalized tetrahedral framework nucleic‐acid nanostructures (OGP‐tFNAs) are prepared; they combine the positive hematopoiesis stimulating effect of OGP and the drug carrying function of tFNAs. The potential of OGP‐tFNAs for hematopoietic stimulation and microenvironment regulation is investigated. It is shown that OGP‐tFNAs can protect bone marrow stromal cells from 5‐fluorouracil (5‐FU)‐induced DNA damage and apoptosis. OGP‐tFNAs pretreatment activates the extracellularly regulated protein kinase signal and downregulates apoptosis‐related proteins. OGP‐tFNAs also alleviate the chemotherapy‐induced inhibition of hematopoiesis‐related cytokine expression, which is crucial for hematopoiesis reconstitution. In conclusion, OGP‐tFNAs can protect hematopoietic cells and their microenvironment from chemotherapy‐induced injuries and myelosuppression, while promoting hematopoiesis regeneration.

## Graphical abstract

Osteogenic growth peptide regulates the bone marrow microenvironment to promote blood cell formation. Tetrahedral nucleic‐acid nanostructures are excellent drug delivery vehicles with additional positive side effects in regenerative medicine.

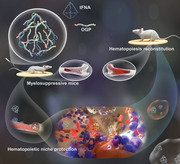

## 1 Introduction

Myelosuppression is a major complication of tumor chemotherapy or radiotherapy. It can cause severe damage to the hemopoietic system and disrupt the bone marrow microenvironment.^[^ ^1^ ^]^ It can also lead to further problems, such as infection, hemorrhage, anemia, or even multiple organ failure.^[^ ^2^ ^]^ Although various drugs have been extensively explored for tumor targeted therapy, traditional chemotherapeutics, such as antimetabolites, antitumor antibiotics, and phytogenic anticarcinogens, are still preferred as anticancer agents. Because these drugs are untargeted, killing both tumor cells and rapidly dividing normal cells including those in bone marrow, most chemotherapeutics cause some degree of myelosuppression.^[^ ^3^ ^]^

Hematopoietic stem cells (HSCs) survive, self‐renew, and proliferate in the bone marrow hematopoietic microenvironment. This microenvironment includes a microvascular system, hematopoiesis‐supportive cells, the extracellular matrix, and various cytokines. An intact hematopoietic microenvironment is essential for hematopoiesis development.^[^ ^4^ ^]^ In addition to damaging HSCs directly, chemotherapy can also damage the hematopoietic microenvironment; this may be the principal cause of long‐term myelosuppression after chemotherapy. Maintaining normal microenvironment function is thus crucial for hematopoietic system reconstitution.^[^ ^5^ ^]^ When a patient receives high‐dose chemotherapy, peripheral blood cannot return to normal levels, even with sufficient HSCs and factors; this suggests that chemotherapy damages the hematopoietic microenvironment. Although the transplantation of hematopoietic factors and hematopoietic stem/progenitor cells can shorten the period of chemotherapy‐induced myelosuppression, hematopoietic and immunologic function still cannot be completely restored.^[^ ^6^ ^]^ Therefore, more attention should be given to the role of chemotherapeutically induced damage to the hematopoietic microenvironment, especially bone marrow stromal cell injuries in myelosuppression treatment.

Currently, the optional therapeutic strategy for myelosuppression utilizes growth factors, e.g., granulocyte‐colony stimulating factor (G‐CSF), granulocyte‐metabotropic colony stimulating factor (GM‐CSF), erythropoietin (EPO), or thrombopoietin (TPO).^[^ ^7^ ^]^ However, growth factors are lineage‐specific and cannot protect the bone marrow from chemotherapy‐induced toxicity.^[^ ^8^ ^]^ A single type of growth factor cannot produce a balanced response in multiple blood cell lines, but must be combined with other factors to reconstruct hematopoietic function.^[^ ^9^ ^]^ Furthermore, growth factor receptors can also be expressed on tumor cells, so their application might lead to malignant growth, tumor progression, or recurrence.^[^ ^10^ ^]^ Therefore, it is important to develop new types of drugs that protect the hematopoietic microenvironment and promote effective hematopoietic reconstitution.

Nucleic‐acid nanomaterials have been extensively applied to tissue engineering and drug delivery.^[^ ^11^ ^]^ Single‐strand DNA can be self‐assembled into a nano‐framework with a specified spatial structure.^[^ ^12^ ^]^ Since it was first reported in 2006,^[^ ^13^ ^]^ DNA origami has shown great potential in biomedicine; it features intelligent self‐assembly, excellent biocompatibility, and structural designability. DNA nanotubes, tetrahedra, and other nanostructures have been used in drug delivery, biosensors, hydrogels, and other applications.^[^ ^14^ ^]^ Although conventional nano‐systems like nanoliposomes, polymer nanoparticles, and inorganic nanoparticles have been reported for drug delivery, induced cytotoxicity and low clearance and biodegradation rates in vivo present major obstacles for their wider biomedical application. In comparison with conventional nano‐systems, DNA as a natural biological nanomaterial has excellent biocompatibility and editability. The size, shape, and spatial structure of DNA can be accurately controlled with rational design. Tetrahedral framework nucleic acids (tFNAs), a special type of DNA nanomaterial with a unique spatial structure, have been employed as promising drug carriers because of their easy functionalization and excellent ability to penetrate cells and tissues;^[^ ^15^ ^]^ they also reportedly promote cell proliferation and alleviate progressive inflammation, showing great potential for regenerative medicine.^[^ ^16^ ^]^ However, the structural instability of DNA nanomaterial after in vivo administration in a complex biological environment represents a major limitation. More generally, nanoparticle–protein interactions complicate the in vivo fate of nanoparticles. Abundant proteins and peptides in the serum might cover the nanoparticles, thus changing the behavior of a nanoparticle‐based drug‐delivery system.^[^ ^17^ ^]^ However, nucleic‐acid–protein hybrid nanostructures show biomedical promise because of their increased structural stability and delivery efficiency.^[^ ^18^ ^]^

Osteogenic growth peptide (OGP) is a homologous peptide isolated from bone marrow that shows a positive response to bone marrow injuries.^[^ ^19^ ^]^ In addition to promoting osteogenesis and bone formation, OGP can promote the hematopoietic response by regulating the bone marrow microenvironment and up‐regulating hematopoiesis‐stimulating factors.^[^ ^20^ ^]^ OGP can improve the overall hematopoietic function with neither immunogenicity nor cytotoxicity. The pre‐adsorption of proteins onto nanostructures has been suggested as one strategy for stabilizing nanoparticle‐based drug delivery systems and reducing the potential influence of additional disturbances in biological fluids; it can increase the bioavailability, predictability, and targeting capacity of the nanostructures after in vivo administration.^[^ ^21^ ^]^ Therefore, advanced OGP adsorption is considered as a promising strategy for nanoparticle surface modification and drug delivery.^[^ ^22^ ^]^

In this study, tFNAs were functionalized with OGP, and the potential effect of OGP‐tFNAs on hematopoiesis stimulation and myelosuppression alleviation was explored. On the one hand, tFNAs could serve as drug carriers for OGP delivery. On the other hand, OGP adsorption could improve the structural stability of tFNAs after systematic administration. OGP‐tFNAs could thus combine the hematopoiesis‐stimulating effect of OGP with the protective effect and drug‐carrying ability of tFNAs. Such a combination of endogenous bioactive peptides with nucleic‐acid nanostructures may offer a new approach to drug delivery.

## 2 Results and Discussion

### 2.1 Synthesis and Characterization of OGP‐tFNAs

**Figure 1:**
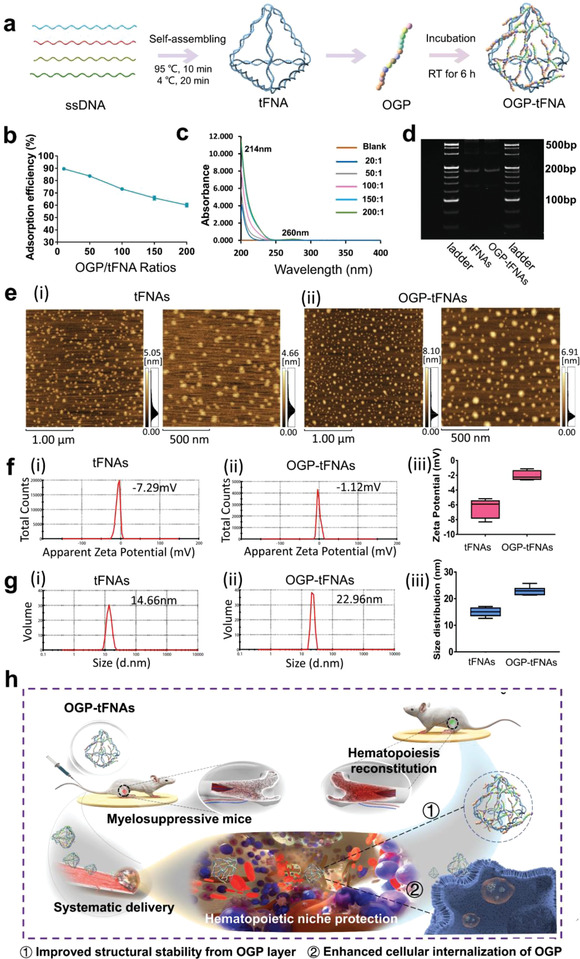
Preparation and characterization of OGP‐tFNAs. a) Schematics of preparation of tFNAs and OGP‐tFNAs. b) The adsorption efficiency of OGP on tFNAs. c) Ultraviolet (UV) absorbance spectra of OGP‐tFNAs with different OGP/tFNA ratios. d) PAGE results for tFNAs and OGP‐tFNAs. e) Morphology of tFNAs and OGP‐tFNAs via AFM observation. f) Zeta potentials of tFNAs and OGP‐tFNAs. g) Size distributions of tFNAs and OGP‐tFNAs. h) Schematics of the systematic delivery and potential effect on hematopoiesis reconstitution of OGP‐tFNAs.

Four specially designed single DNA strands were successfully self‐assembled into tFNAs (**Figure** **1**a, Table S1, Supporting Information). According to the polyacrylamide gel electrophoresis (PAGE) result, the OGP‐tFNAs were prepared with relatively high productivity (Figure S1d, Supporting Information). The adsorption efficiency of OGP decreased from ≈90% to 60% as the OGP/tFNA ratio increased (Figure 1b), whereas the tFNAs loading capacity increased (Figure S2b, Supporting Information). OGP consists of fourteen basic and neutral amino acids with the absorbance at 214 nm (Figure S1b,c, Supporting Information); its theoretical isoelectric‐point pH is 11.38. In the tFNAs self‐assembling system, the pH of the Tris‐maleate (TM) buffer was 8.0; the OGP was positively charged in it (Figure S1c, Supporting Information), which contributed to the adsorption of OGP onto the tFNAs via electrostatic incorporation. Therefore, more OGP adsorption could be detected with the increase of OGP/tFNA ratios (Figure 1c). The combination of OGP and tFNAs could also be seen from the band position and intensity changes (Figure 1d, Figure S2a, Supporting Information). Morphological changes were furtherly detected after OGP adsorption. In atomic‐force micrographs, OGP‐tFNAs presented a rounder shape in comparison with bare tFNAs (Figure 1e); this indicated OGP layer formation on the tFNAs.

Dynamic light scattering (DLS) measurements showed that the tFNAs had a zeta potential of ≈−7 mV (Figure 1f) and an average size of ≈15 nm (Figure 1g). After OGP adsorption, the zeta potential increased to ≈−1 mV (Figure 1f) and the average OGP‐tFNAs size was ≈23 nm (Figure 1g), further proving the adsorption of OGP. According to these results, OGP layers formed on the tFNAs, which might improve their structural stability after systematic delivery of OGP‐tFNAs (Figure 1h). Then, the drug‐release kinetic was investigated to detect the OGP release from tFNAs. The results suggested an abrupt release at 30 min, and the release ratio remained relatively stable at a relative lower speed (Figure S2c, Supporting Information). To further investigate the stability of OGP‐tFNAs in different concentrations of serum, 10%, 5%, and 2% fetal bovine serum (FBS) were used for stability testing. As shown in Figure S2d (Supporting Information), OGP‐tFNAs remained stable for ≈12–24 h in 2% FBS and 6–9 h in 10% FBS. The stability decreased with increasing FBS concentration.

### 2.2 Cellular Uptake and Cell Viability

Bone marrow stromal cells are crucial supportive cells in the hematopoietic microenvironment; they participate in the homing, adhesion, self‐renewal, proliferation, and multilineage differentiation of HSCs via the direct contact and secretion of hematopoietic regulatory factors.^[^ ^23^ ^]^ In this study, OP9 bone marrow stromal cells were employed to explore the potential biological effect of OGP‐tFNAs on hematopoiesis stimulation.

**Figure 2:**
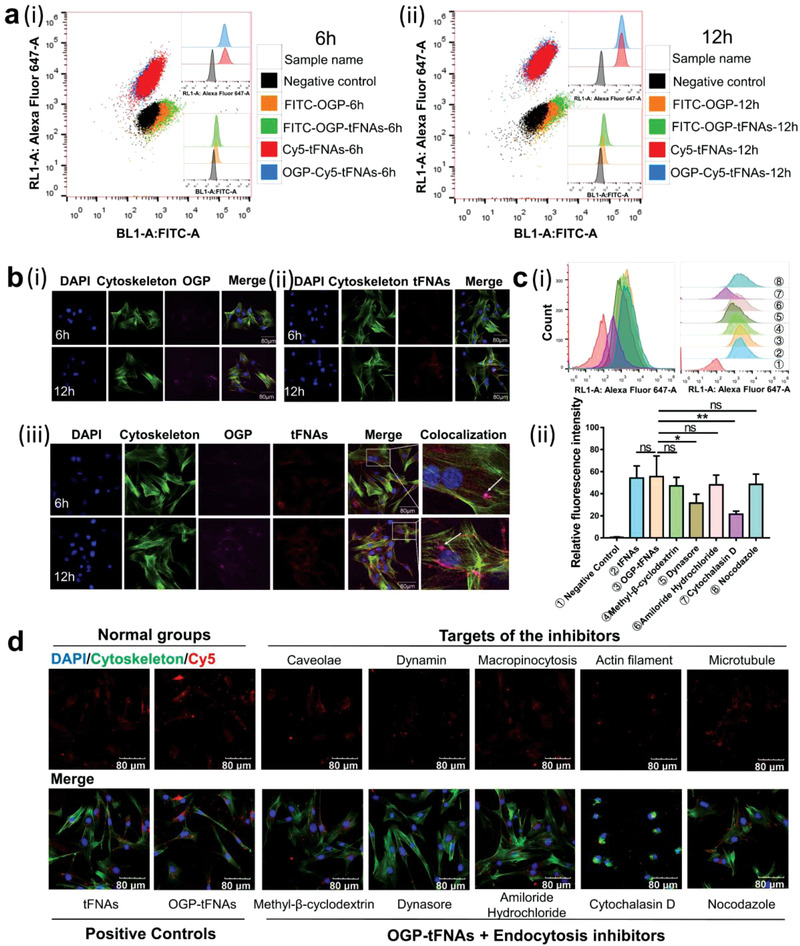
Cellular uptake and internalization mechanisms of OGP‐tFNAs. a) Cellular uptake of OGP, tFNAs, and OGP‐tFNAs at 6 and 12 h via flow cytometric analysis. b) (i) Confocal fluorescence microscopy images of the cellular internalization of FITC‐labeled OGP; the OGP channel was stained purple to allow optical distinction from the cytoskeleton; (ii) Confocal fluorescence microscopy images for cellular internalization of Cy5‐labeled tFNAs. (iii) The distribution and colocalization of OGP and tFNAs in OP9 cells. c) (i) Flow cytometric analysis for internalization mechanism of OGP‐tFNAs. Five inhibitors were used to investigate the inhibition of related endocytosis pathways; (ii) Statistical analysis for the average fluorescence intensity; data are presented as mean ± SD (*n* = 3): *p < 0.05, **p < 0.01. d) Confocal microscopy observation of the cellular uptake of OGP‐tFNAs after treatment with different endocytosis inhibitors.

To study the potential influences of OGP adsorption on the cellular uptake of tFNAs and vice versa, tFNAs and OGP were fluorescently labeled. The cellular internalization of OGP‐tFNAs was detected via flow cytometric analysis. For simplex OGP, the cellular uptake and fluorescence intensity were low at both 6 and 12 h, but the cellular uptake increased after incorporation with tFNAs (**Figure** **2**a(i)). For tFNAs, however, adsorption of OGP did not significantly change the cellular uptake of tFNAs at either 6 or 12 h (Figure 2a). The intracellular distribution was also detected via confocal fluorescence microscopy. To distinguish the phalloidin‐stained cytoskeleton from OGP labeled with fluorescein isothiocyanate (FITC), the OGP‐FITC channel was colored purple after confocal fluorescence image capture (Figure 2b). More OGP/tFNAs were internalized by OP9 cells at 12 h than that at 6 h. According to the colocalization images, OGPs and tFNAs were located at the same positions in the cytoplasm, further demonstrating the incorporation of OGP with the tFNAs (Figure 2b(iii)).

The endocytosis mechanism of OGP‐tFNAs was further investigated. According to the literature, the endocytosis mechanisms can be classified as phagocytosis, pinocytosis, and receptor‐mediated endocytosis.^[^ ^24^ ^]^ The internalization of nanoparticles mainly depends on receptor‐mediated endocytosis, which includes clathrin/caveolae‐mediated endocytosis, and clathrin/caveolae‐independent endocytosis.^[^ ^25^ ^]^ The endocytosis mechanism of OGP‐tFNAs was explored accordingly. Five different inhibitors were used to inhibit the clathrin, caveolae macropinocytosis, microfilaments, and microtubules separately, as shown in Table S2 (Supporting Information). The internalization of OGP‐tFNAs after treatment with different inhibitors was detected via flow cytometric analysis and confocal fluorescence microscopy observation. According to the results (Figure 2c,d), Dynasore and Cytochalasin D showed more significant inhibition ratios, which suggested that clathrin mainly contributed to the internalization of OGP‐tFNAs and actin was also involved in the endocytosis process. Although there was no significant statistical difference for the other inhibitors, the internalization ratios also decreased in different degrees, which indicated that caveolae, tubulins, and macropinocytosis were also involved in the internalization of OGP‐tFNAs.

It has been reported that tFNAs could promote the proliferation of stem, epithelial, and mesenchymal cells.^[^ ^26^ ^]^ Their influence on the proliferation of OP9 cells was investigated in this study. As shown in Figure S3a (Supporting Information), 250 nm tFNAs had the most significant proliferation‐promoting effect on OP9 cells, this concentration was selected for use in later experiments. OGP is reportedly a mitogen;^[^ ^27^ ^]^ this characteristic could promote the proliferation of osteoblasts and bone marrow HSCs. However, the biological effect of OGP on bone marrow cells is largely unexplored. In this study, OGP was combined with 250 nm tFNAs with OGP/tFNA ratios from 5:1 to 200:1 (Figure S3b, Supporting Information). Because OGP‐tFNAs with a 100:1 OGP/tFNA ratio was found to have the most significant promoting effect on the viability of OP9 cells, this ratio was selected for use in later experiments. Furtherly, OGP‐tFNAs had a stronger promoting effect on OP9 cell viability than simplex OGP and tFNAs. Therefore, the combined delivery of OGP and tFNAs showed better bioactivity than single‐component delivery.

### 2.3 OGP‐tFNAs Alleviation of Chemotherapeutically Induced Bone Marrow Stromal Cell Apoptosis

**Figure 3:**
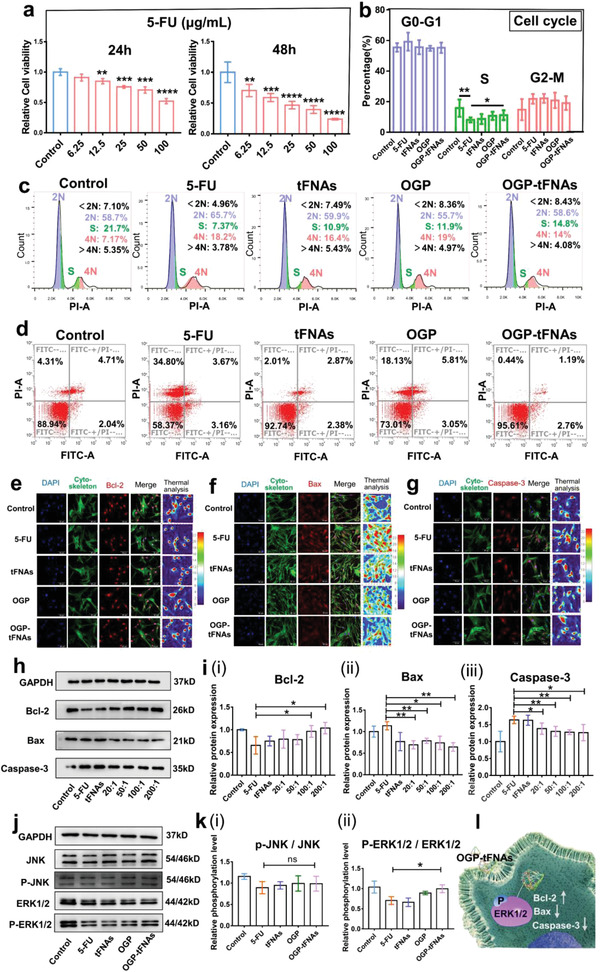
The protective effect of OGP‐tFNAs on OP9 cells from 5‐FU induced injury. a) CCK‐8 assays to detect the inhibitory effect of 5‐FU (*n* = 6). b,c) Cell cycle analysis after 12 h of pretreatment with tFNAs, OGP, and OGP‐tFNAs, followed by 24 h 5‐FU treatment (*n* = 3). d) Flow cytometric analysis of cell apoptosis after FITC and propidium iodide staining (PI) staining. e) Immunofluorescent staining for Bcl‐2. f) Immunofluorescent staining for Bax. g) Immunofluorescent staining for Caspase‐3. h) Western blot analysis (WB) results for Bcl‐2, Bax, and Caspase‐3 expression. i) Semiquantitative analysis for WB results of Bcl‐2, Bax, and Caspase‐3 (*n* = 3). j) WB results for JNK, P‐JNK, ERK1/2, and p‐ERK1/2. k) Semi‐quantitative analysis for WB results of P‐JNK and P‐ERK1/2 (*n* = 3). l) Illustration of the protective effect of OGP‐tFNAs via ERK1/2 signal activation. Data are presented as mean ± SD: *p < 0.05, **p < 0.01, ***p < 0.001, and ****p < 0.0001.

To determine the proper concentration for the in vitro model of bone marrow stromal cell injuries, OP9 cells were treated with the commonly used damage‐inducing agent 5‐FU for 24 and 48 h in concentrations from 6.25 to 100 µg mL^−1^. As shown in **Figure** **3**a, 5‐FU treatment obviously inhibited the cell viability of OP9 cells in a dose‐dependent manner. At 48 h, 25 µg mL^−1^ 5‐FU showed the median inhibitory effect on OP9 cells.

OP9 cells were pretreated with OGP‐tFNAs for 12 h; then, for an additional 24 h, they were treated with 25 µg mL^−1^ 5‐FU. The same treatments were also performed on the simplex OGP and tFNAs groups. To eliminate the possible influence of components of the TM buffer on the control and 5‐FU groups, equal volumes of TM buffer were added to their culture media. After the treatments, the samples were collected for cell cycle detection via flow cytometric analysis (Figure 3b,c). In the chemotherapy group, 5‐FU obviously inhibited the proliferation of OP9 cells and reduced the percentage of S‐stage cells, whereas an increased percentage of S‐stage cells was observed in the OGP‐tFNAs pretreatment groups.

The apoptosis of OP9 cells was also detected by flow cytometric analysis (Figure 3d). In the 5‐FU group, the number of dead OP9 cells approached ≈30%. Although OGP and tFNAs pretreatment reduced cell apoptosis to some extent, OGP‐tFNAs showed the strongest protective effect. Apoptosis related proteins (including Caspase‐3, Bcl‐2, and Bax) provided another way to measure the protective effect of OGP‐tFNAs. 5‐FU treatment downregulated the expression of Bcl‐2, and OGP‐tFNAs pretreatment alleviated the downregulation effect (Figure 3e,h,i). Conversely, the expression of Caspase‐3 and Bax decreased with increasing OGP/tFNA ratio (Figure 3f,g,i), in agreement with the cytometric analysis shown in Figure 3d. It is well known that Bcl‐2 can inhibit cell death and enhance resistance to most DNA‐damaging factors, including chemotherapeutic drugs. Therefore, OGP‐tFNAs counteracted the 5‐FU‐induced Bcl‐2 decrease, while Bcl‐2 increased the resistance of OP9 cells to 5‐FU induced injuries.

The mitogen‐activated protein‐kinase (MAPK) signaling pathway is closely related to cell proliferation, differentiation, and apoptosis. As important members of the MAPK family, extracellular regulated protein kinase (ERK) and c‐Jun N‐terminal kinase (JNK) can respond to a variety of extracellular stimuli, including stress stimulation, mitogen, and growth factors, and participate in regulating cell proliferation and apoptosis, DNA damage repair, and other cell biological reactions.^[^ ^28^ ^]^ Activation of the ERK signaling pathway can promote cell proliferation, while the activation of JNK signaling pathways is related to cell apoptosis. As shown in Figure 3j,k, 5‐FU treatment significantly inhibited the activation of ERK signals. By contrast, OGP‐tFNAs reversed the inhibitory effect of 5‐FU on ERK activation, in accordance with the expression of downstream Bcl‐2 (Figure 3h). However, the JNK signaling pathways did not seem to be significantly affected. These results suggested that ERK pathways might be more important for the protective effect of OGP‐tFNAs on chemotherapy‐induced OP9 cell injuries (Figure 3l).

### 2.4 Proliferative Ability Protection and Hematopoietic Cell Factor Expression Maintenance in OP9 Cells

**Figure 4:**
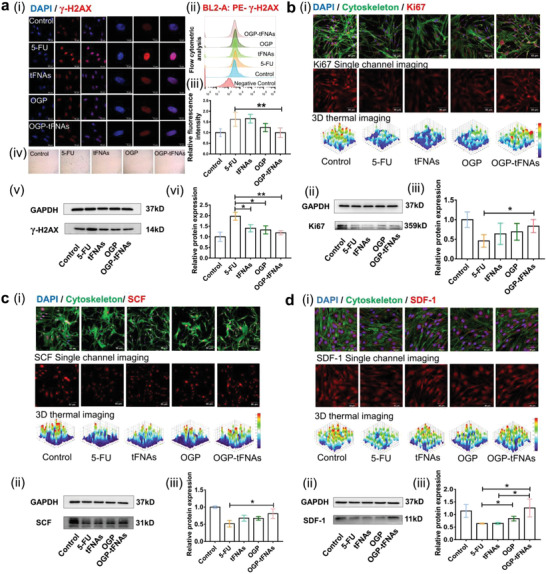
DNA damage, cell proliferation, and hematopoiesis related protein expression in OP9 cells. a) (i) Immunofluorescent staining for *γ*‐H2AX expressions to mark the DNA damage; (ii) Flow cytometric analysis for *γ*‐H2AX; (iii) Statistical analysis for flow cytometric results of *γ*‐H2AX (*n* = 5); iv) *β*‐Galactosidase staining for cell senescence detection; v) WB results of *γ*‐H2AX; vi) Semiquantitative analysis of WB results for *γ*‐H2AX expression (*n* = 3). b) Immunofluorescent staining, WB results, and semiquantitative analysis of WB results for Ki‐67 expression (*n* = 3). c) Immunofluorescent staining, WB results, and semiquantitative analysis of WB results for SCF expression (*n* = 3). d) Immunofluorescent staining, WB results, and semiquantitative analysis of WB results for SDF‐1 expression (*n* = 3). Data are presented as mean ± SD: *p < 0.05, **p < 0.01.

DNA damage is one of the major injurious effects of irradiation and most chemotherapeutic drugs; it can cause cell senescence and stagnation. Single‐ or double‐strand breaks can result from 5‐FU‐induced DNA base‐pair mismatch and replication stagnation. Increased expression of *γ*‐H2AX, a biomarker for DNA damage, has been observed in chemotherapy‐injured cells.^[^ ^29^ ^]^ In this study, increased *γ*‐H2AX expression was found in the 5‐FU group compared with the control group; this indicated that DNA damage occurred in OP9 cells after 5‐FU treatment. However, the *γ*‐H2AX level of the OGP‐tFNAs group was lower than that in other groups, indicating that OGP‐tFNAs pretreatment reduced DNA damage (**Figure** **4**a). To demonstrate the protective effect of OGP‐tFNAs against the 5‐FU‐induced senescence of OP9 cells, *β*‐galactosidase staining was used to mark senescent cells (Figure 4a(iv)). As anticipated from the *γ*‐H2AX levels, significantly fewer senescent cells were observed in the OGP‐tFNAs pretreatment group than in the 5‐FU group. The alleviation of 5‐FU‐induced OP9‐cell senescence showed that OGP‐tFNAs protect DNA integrity and support normal replication maintenance.

Maintaining the self‐renewal and proliferative abilities of hematopoiesis‐related cells is an important strategy for myelosuppression prevention and treatment.^[^ ^30^ ^]^ Ki67 is a mitosis‐associated antigen that is indispensable for cell proliferation. Therefore, Ki67 expression was measured to study the proliferative activity of OP9 cells (Figure 4b). The overall expression of Ki67 for the OGP‐tFNAs pretreatment group was higher than in other groups, which indicated that OGP‐tFNAs maintain the proliferative ability of OP9 cells.

The hematopoietic growth factors (HPGFs) secreted by bone marrow stromal cells also play crucial roles in the mobilization and recovery of hematopoietic function after chemotherapy and bone marrow transplantation.^[^ ^31^ ^]^ Stem cell factor (SCF), an important HPGF,^[^ ^32^ ^]^ has been shown to bind with C‐Kit receptors and promote pluripotent stem‐cell proliferation and hematopoiesis regeneration.^[^ ^33^ ^]^ After chemotherapy, the normal secretory and supportive function of stromal cells can be reduced.^[^ ^34^ ^]^ In the present study, 5‐FU treatment reduced the SCF production in OP9 cells by ≈50% (Figure 4c); with OGP‐tFNAs treatment protecting the secretory ability, the SCF level remained at ≈80% of its normal value.

Bone marrow stromal cells also support hematopoiesis by producing chemokines that promote the homing of HSCs. An example is stromal cell derived factor‐1 (SDF‐1), which was first cloned from mouse bone marrow stromal cells by Nagasawa et al.;^[^ ^35^ ^]^ they found that SDF‐1‐deficient mice failed to develop lymphocytes and bone marrow myeloid cells.^[^ ^36^ ^]^ It has since been understood that SDF‐1 provides the physiological signal for the homing of hematopoietic cells to bone marrow and is thus critical to their subsequent colonization, maintenance, survival, and development in the bone marrow microenvironment. SDF‐1 deficiency can reportedly lead to bone marrow hematopoiesis failure; it also disrupts the homeostasis of hematopoietic stem/progenitor cells.^[^ ^37^ ^]^ As shown in Figure 4d, 5‐FU treatment decreased the expression of SDF‐1 in OP9 cells, but pretreatment with OGP‐tFNAs offered protection from this effect. The OGP‐tFNAs protected SDF‐1 expression may prove important for hematopoiesis reconstitution.

### 2.5 Bone Marrow Protection and Myelosuppression Alleviation in Mice

**Figure 5:**
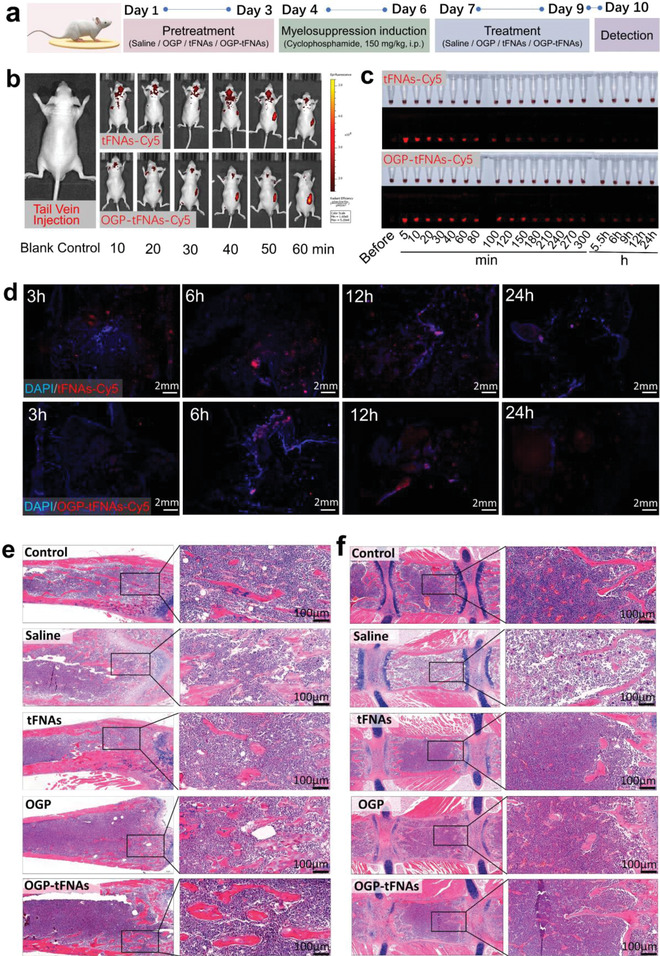
In vivo administration and protective effect of OGP‐tFNAs on the hematopoietic function in mice. a) Timeline for saline, tFNAs, OGP, and OGP‐tFNAs pretreatment and myelosuppression induction. b) In vivo distribution of tFNAs and OGP‐tFNAs after tail vein injection. Cy5 was used to label tFNAs and OGP‐tFNAs. c) Plasma pharmacokinetics of tFNAs and OGP‐tFNAs. d) Confocal microscopy images for bone marrow smears of femur at 3, 6, 12, and 24 h after tail vein injection of tFNAs and OGP‐tFNAs. e) H&E staining for femur marrow. f) H&E staining for sternum marrow.

OGP‐tFNAs were delivered via tail‐vein injection to mouse myelosuppression models. The timeline of drug delivery and treatment in the mice is shown in **Figure** **5**a. OGP‐tFNAs were administrated for three days prior to myelosuppression induction for advanced hematopoiesis mobilization. The tFNAs were labeled with Cy5 to track their in vivo distribution; they mainly accumulated in the kidney (Figure 5b). The fluorescence intensity gradually increased in the first 50 min and then started decreasing, reflecting progressive accumulation and clearance in the kidney. OGP adsorption changed the tFNAs accumulation time: the fluorescence intensity continuously increased for 60 min, indicating delayed clearance in the kidney and prolonged in vivo distribution (Figure 5b). Pharmacokinetic experimentation was then performed to detect the in vivo metabolism. According to the results, the half‐life period was ≈60 min for OGP‐tFNAs and ≈30–40 min for bare tFNAs, which also suggested that OGP adsorption suppressed the clearance rate of tFNAs (Figure 5c and Figure S4a, Supporting Information).

The body weight was also recorded. Although there was no obvious statistical difference between the OGP, tFNAs, and OGP‐tFNAs groups, the overall bodyweight of mice in the OGP‐tFNAs group was relatively higher (Figure S4b, Supporting Information). Bone marrow is the major place where hematopoiesis takes place, and the histological changes in bone marrow directly reflect hematopoietic function. To investigate whether the OGP‐tFNAs could arrive in the bone marrow via tail vein injection, the femurs were collected for bone marrow smearing and confocal microscopy observation at 3, 6, 12, and 24 h after tail‐vein injection. As shown in Figure 5d, the OGP‐tFNAs successfully arrived in the bone marrow, which contributed to the subsequent biological responses.

Under physiological conditions, the reticular bone marrow cavity is filled with various parenchymal cells, including HSCs, progenitors, and mature blood cells. In the control group (Figure 5e,f), the bone marrow cells showed active growth and filled ≈80% of the bone marrow cavity. Myelosuppression induction with a chemotherapeutic drug like cyclophosphamide severely inhibits the proliferation and self‐renewal of bone marrow cells, such that they fill only ≈20–30% of the bone marrow cavity. For the saline pretreatment and myelosuppression induction group, the number of nucleated cells in bone marrow from the femur and sternum was significantly reduced, and the bone marrow was filled with adipose tissue. In the OGP, tFNAs, and OGP‐tFNAs groups, the bone marrow showed active proliferation, and abundant blood cells were observed. In addition, the blood was also monitored for important peripheral hematological parameter detection, including the white blood cell count (WBC), hemoglobin level (HGB), lymphocyte count (LY), and monocyte percentage (Mon%). As the results suggested, the OGP‐tFNAs group showed better cell counts and percentages for the peripheral blood cells (Figure S4c, Supporting Information), which was consistent with the H&E staining results of the femurs/sternums and suggested better protection for the hemopoietic system against chemotherapy injury (Figure 5e,f).

The spleen plays a major role in hematopoiesis in the early embryonic stage as a blood reservoir and immune organ; however, its hematopoietic function is mainly replaced by that of bone marrow after birth. The normal structure of the spleen can be damaged by chemotherapeutic drugs. The spleen index of the OGP‐tFNAs group was higher than that of the other groups, once again suggesting a protective effect of the OGP‐tFNAs toward the hematopoietic and immune systems (Figure S4d, Supporting Information). H&E staining of the spleen showed that, for the saline group, the white pulp and germinal center were reduced, and the number of megakaryocytes and lymphocytes decreased significantly. These results suggested that the drugs did obvious damage to the spleens of the myelosuppressed mice. Compared with those of the saline group, the spleen structures of the intervened groups remained relatively normal. Especially in the OGP‐tFNAs group, the white‐pulp structure and germinal center were clearer, and abundant lymphocytes were observed in the splenic cord (Figure S4e, Supporting Information). It is generally thought that the spleen can compensate for reduced bone marrow hematopoietic function. Therefore, changes in the spleen weight and index can indirectly reflect hematopoietic ability. Furtherly, the safety evaluation of tFNAs and OGP‐tFNAs was performed in healthy ICR mice, and no obvious toxicity was observed for both groups at day 5 and day 10 (Figure S5, Supporting Information).

### 2.6 Hematopoietic Cells and Cell Factor Expression for Hematopoiesis Regeneration in Mice Bone Marrow

After the concept of “niche” was first presented by Schofield,^[^ ^38^ ^]^ studies on hematopoiesis have not been confined to HSCs alone, but have included the whole hematopoietic microenvironment.^[^ ^4a,c^ ^]^ The microenvironment regulates the hematopoietic cells mainly via direct cell–cell contact, hematopoietic‐stimulating‐factor secretion, and chemokine secretion for HSC homing.^[^ ^39^ ^]^ For the treatment of myelosuppression, the priorities are maintaining cell viability and protecting bone marrow cells from chemotherapy‐induced injuries.^[^ ^40^ ^]^

**Figure 6:**
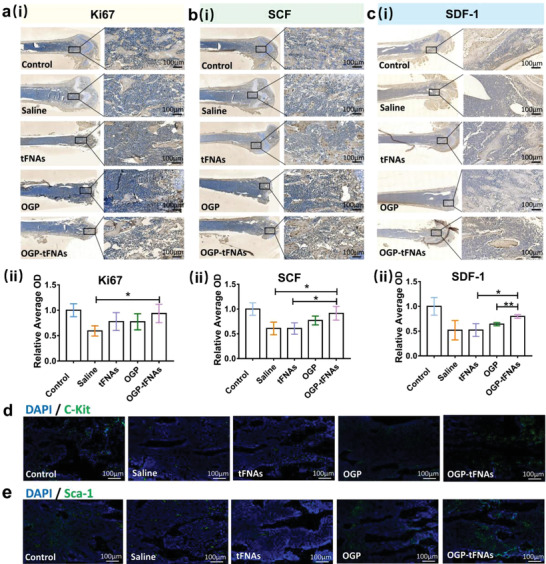
Immunohistochemical and immunofluorescent staining for proliferation and hematopoiesis related proteins in mice femurs. a) (i) Immunohistochemical staining for Ki67; (ii) Semiquantitative analysis Ki‐67 expression (*n* = 3). b) (i) Immunohistochemical staining for SCF; (ii) Semiquantitative analysis SCF expression (*n* = 3). c) (i) Immunohistochemical staining for SDF‐1; (ii) Semiquantitative analysis SDF‐1 expression (*n* = 3). d) Immunofluorescent staining for C‐Kit‐positive cells. e) Immunofluorescent staining for Sca‐1‐positive cells. Data are presented as mean ± SD: *p < 0.05, **p < 0.01.

In this study, cell proliferation was detected via Ki67 immunohistochemical staining. As shown in **Figure** **6**a, the percentage of Ki67‐positive cells was reduced in comparison with the control group. The overall proliferation and cell growth status in the OGP‐tFNAs group was better than those in the other groups, which indicated that OGP‐tFNAs help to maintain the self‐renewal and proliferation abilities of bone marrow cells. The OGP‐tFNAs group also consistently showed more SCF and SDF‐1 expression in femoral bone marrow (Figure 6b,c).

Further, the expressions of C‐Kit and Sca‐1 in the bone marrow were also detected, as these are important HSC markers. The interaction between HSC and the hematopoietic microenvironment is regulated by growth factors, chemokines, and adhesion factors, which maintain balance between the self‐renewal and differentiation states.^[^ ^41^ ^]^ The SCF‐Kit ligand/receptor system is an important regulation pathway that promotes the self‐renewal and adhesion of HSCs to the microenvironment.^[^ ^42^ ^]^ An SCF receptor, C‐Kit is normally expressed on hematopoietic stem/progenitor cells;^[^ ^43^ ^]^ blocking C‐Kit can lead to a decrease in the number of HSCs.^[^ ^44^ ^]^ Sca‐1 is the other marker of mouse HSCs; Sca‐1‐positive cells can be found in the bone marrow, spleen, and peripheral blood, which also includes some progenitor cells in addition to HSCs.^[^ ^45^ ^]^ Zhou et al.^[^ ^46^ ^]^ reported that the transplant of Sca‐1‐positive hematopoietic stem/progenitor cells could reconstruct the hematopoietic function of mice suffering from lethal doses of radiation. Ito et al.^[^ ^47^ ^]^ reported decreased multipotential differentiation ability of bone marrow cells in Sca‐1 deficient mice and unsatisfying survival rates after the transplantation of Sca‐1‐negative cells to wild‐type mice after lethal irradiation. These studies indicated that C‐Kit and Sca‐1 are crucial in the self‐renewal and development of HSCs, and that C‐Kit‐positive and Sca‐1‐positive cells are essential for hematopoiesis reconstitution. As shown in Figure 6d,e, C‐Kit and Sca‐1 in the OGP‐tFNAs group retained higher expression, which indicated the possible protective effect of OGP‐tFNAs for HSCs in the bone marrow. The hematopoietic function relies on a complete microenvironment system; chemotherapy induced damage to the bone marrow could lead to the depletion of hematopoietic pools and disturb the normal hematopoietic microenvironment.^[^ ^48^ ^]^ Therefore, protecting the hematopoietic microenvironment from chemotherapy‐induced damage is critical for myelosuppression alleviation and hematopoietic reconstitution.

## 3 Conclusions

In this study, the protective effect of OGP‐tFNAs against chemotherapy‐induced myelosuppression was demonstrated. OGP‐tFNAs were prepared to combine the hematopoiesis‐stimulating effectiveness of OGP and the drug‐carrying effectiveness of tFNAs. OGP‐tFNAs pretreatment activated the ERK signal and reduced 5‐FU‐induced DNA damage and apoptosis, thus maintaining the normal function of OP9 cells. Furthermore, the expression of the SCF and the chemokine SDF‐1 was also higher after OGP‐tFNAs pretreatment both in vitro and in vivo. Therefore, OGP‐tFNAs show promise for myelosuppression alleviation and hematopoiesis reconstitution. More generally, the combination of bioactive peptides and nucleic‐acid nanomaterials may provide a new strategy for advanced drug delivery. For future applications, tFNAs could be modified with hematopoietic‐cell‐targeted aptamers and therapeutic oligonucleotides drugs for more targeted and stronger hematopoiesis promotion.

## 4 Experimental Section

### Animal Experiment

The animal experiment was approved by the Ethics Committee of West China Hospital of Stomatology, Sichuan University (Approval Number: WCHSIRB‐D‐2019‐102) and conducted according to the ARRIVE guidelines. 6–8 weeks male ICR mice (Dossy Experimental Animals Co, Ltd., Chengdu, China) were raised in specific pathogen‐free (SPF) environment with stable temperature and 12 h light/dark cycle.

### Statistical Analysis

All quantitative data were presented as mean ± standard deviations (mean ± SD), and the data were statistically analyzed using one‐way analysis of variance (ANOVA) among multiple groups or two‐tailed unpaired student's *t*‐test between two groups. The statistical analysis was performed using GraphPad Prism 7.0 (GraphPad Software Inc., San Diego, CA, USA). Data significances were shown with *p* value: *p < 0.05, **p < 0.01, ***p < 0.001, and ****p < 0.0001.

Additional detailed materials and methods can be found in the Supporting Information.

## Conflict of Interest

The authors declare no conflict of interest.

## Supporting information

Supporting Information

## Data Availability

The data that support the findings of this study are available from the corresponding author upon reasonable request.
