# Supplementary material for: Myelosuppression Alleviation and Hematopoietic Regeneration by Tetrahedral‐Framework Nucleic‐Acid Nanostructures Functionalized with Osteogenic Growth Peptide
Source: Adv Sci (Weinh). 2022 Jul 26;9(27):2202058. doi: 10.1002/advs.202202058 (PMC9507378; doi:10.1002/advs.202202058)
Supplement: Supplementary file 1 — Supporting Information [file ADVS-9-2202058-s001.pdf]

ADVANCED SCIENCE
Open Access

Supporting Information

for *Adv. Sci.*, DOI 10.1002/advs.202202058

Myelosuppression Alleviation and Hematopoietic Regeneration by Tetrahedral-Framework Nucleic-Acid Nanostructures Functionalized with Osteogenic Growth Peptide

*Tianxu Zhang, Mi Zhou, Dexuan Xiao, Zhiqiang Liu, Yueying Jiang, Maogeng Feng, Yunfeng Lin and Xiaoxiao Cai**

Supporting Information

Myelosuppression Alleviation and Hematopoietic Regeneration by Tetrahedral-Framework Nucleic-Acid Nanostructures Functionalized with Osteogenic Growth Peptide

Tianxu Zhang[1], *Mi Zhou*[1], *Dexuan Xiao*[1], *Zhiqiang Liu*[1], *Yueying Jiang*[1], *Maogeng Feng*[2], *Yunfeng Lin*[1] *and Xiaoxiao Cai*[*1]

[1] State Key Laboratory of Oral Diseases, National Clinical Research Center for Oral Diseases, West China Hospital of Stomatology, Sichuan University, Chengdu 610041, P. R. China

[2] Department of Oral and Maxillofacial Surgery, The Affiliated Stomatology Hospital of Southwest Medical University, Luzhou 646000, P. R. China

*Corresponding author: Xiaoxiao Cai

E-mail address: xcai@scu.edu.cn

Tel/Fax: 86-28-85503487

State Key Laboratory of Oral Diseases, National Clinical Research Center for Oral Diseases, West China Hospital of Stomatology, Sichuan University, Chengdu 610041, P. R. China.

Supplementary Figures and Tables

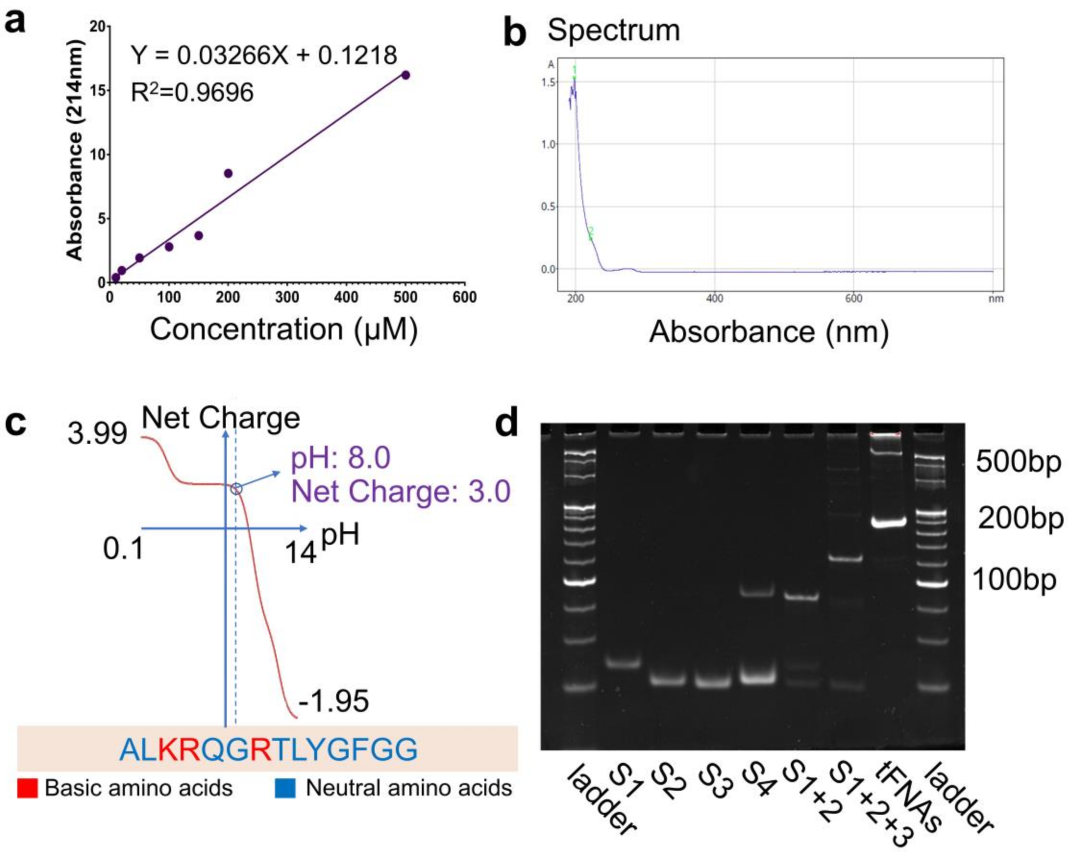

Figure S1. Characterization of OGP and tFNAs. a) Standard curve of OGP. b) UV absorbance spectra of OGP. c) Isoelectric point and amino acid composition of OGP. d) PAGE result for tFNAs production.

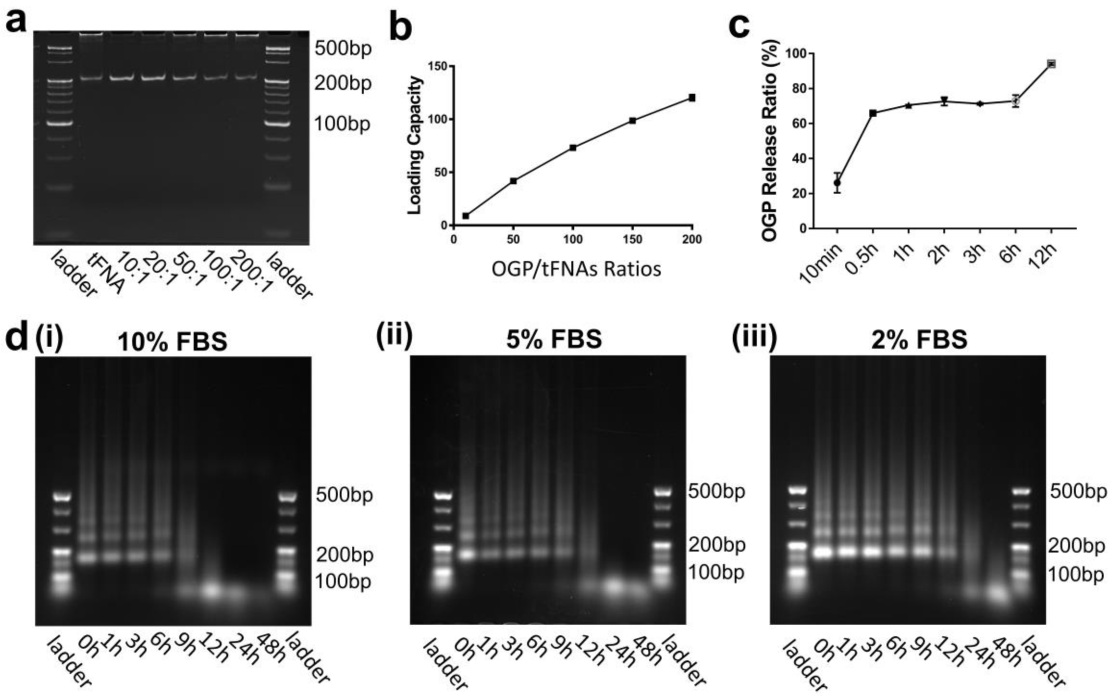

Figure S2. a) PAGE results for OGP-tFNAs with different OGP/tFNA ratios. b) The loading capacity of tFNAs with OGP, which is referred to the molar ratio of adsorbed OGP and tFNAs. c) In vitro OGP release kinetics. d) Agarose gel electrophoresis for the stability testing of OGP-tFNAs in different concentrations of FBS from 0h to 48h at 37℃.

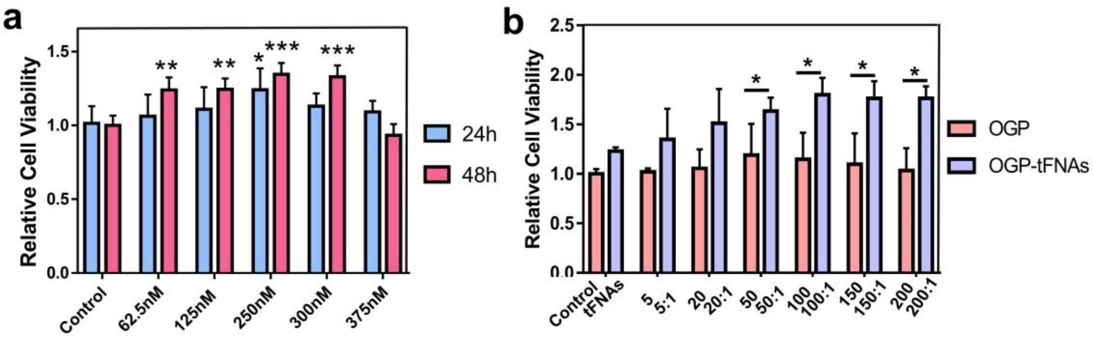

Figure S3. Effect of OGP, tFNAs and OGP-tFNAs on the cell viability of OP9 cells. a) The effect of tFNAs. b) The combination effect of OGP and tFNAs.

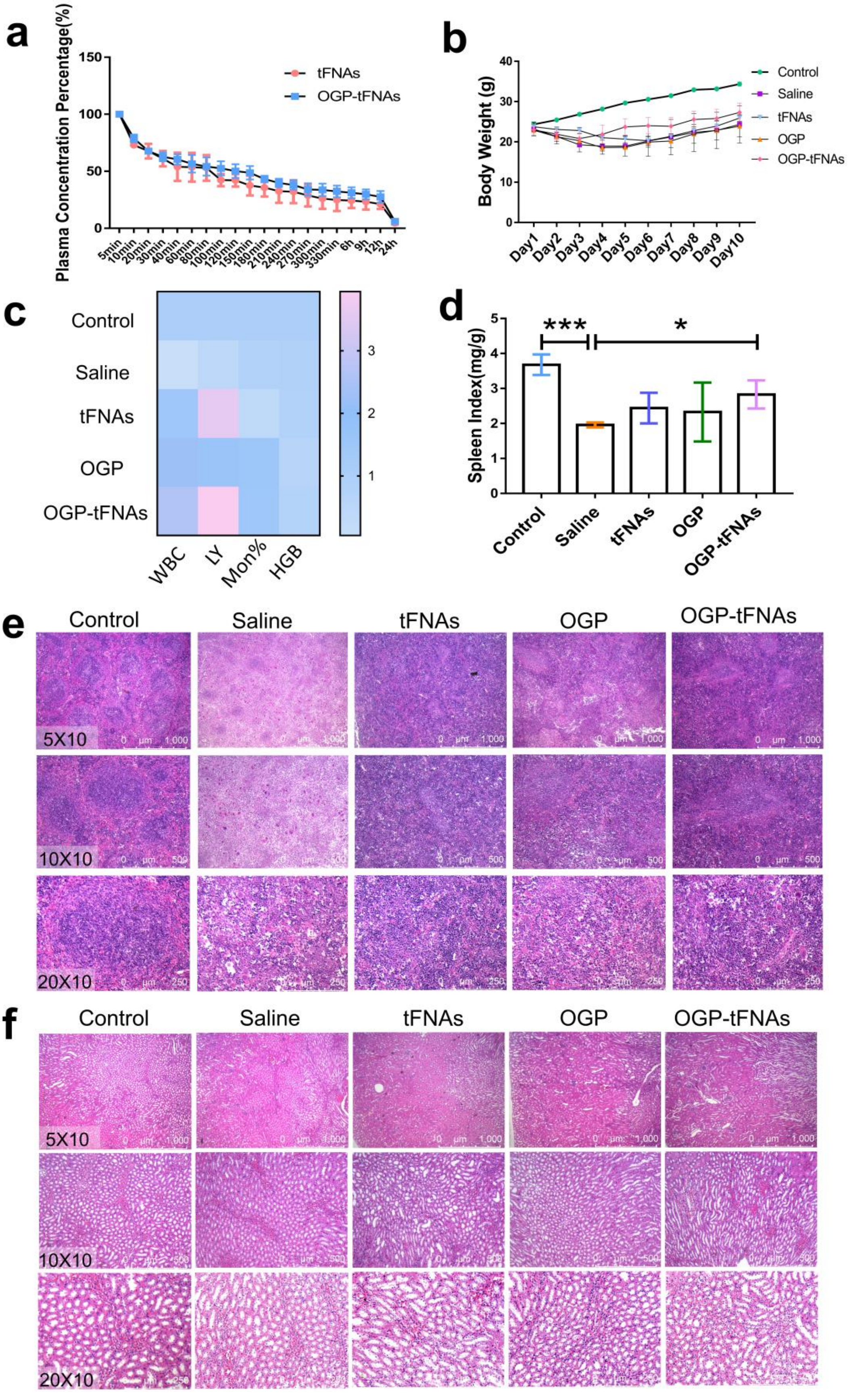

Figure S4. Plasma pharmacokinetics and protective effects of OGP-tFNAs. a) Plasma pharmacokinetics. b) Body weight changes after myelosuppression induction and

different treatment. c) Heat map of the relative changes of white blood cell count (WBC), lymphocyte count (LY), and monocyte percentage (Mon%) and hemoglobin level (HGB). d) The spleen index of mice in different groups. Data are presented as mean ± SD (n = 4): *p <0.05, ***p < 0.001. e) H&E staining for spleen. f) H&E staining for kidney.

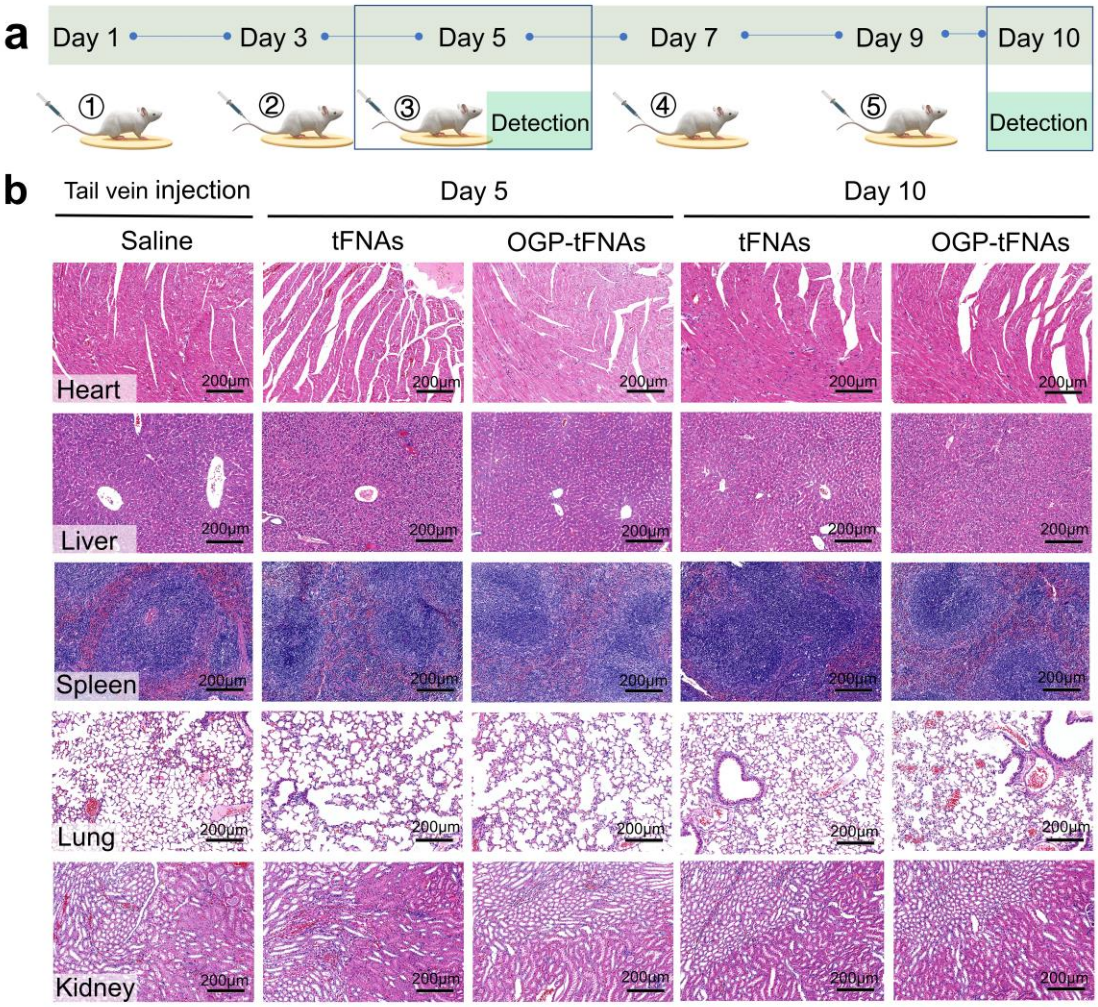

Figure S5. Safety evaluation of tFNAs and OGP-tFNAs. a) Timeline for systematic delivery of tFNAs and OGP-tFNAs. b) H&E staining for major organs.

Table S1. Base sequence of ssDNA for tFNAs self-assembling

SSDNA	Sequences
S1	5′- ATTTATCACCCGCCATAGTAGACGTATCACCAGGCAGTTG GACGAACATTCCTAAGTCTGAA-3′
S2	5′- ACATGCGAGGGTCCAATACCGACGATTACAGCTTGCTACACGATTCAGACTTAGGAATGTTCG-3′

S3	5′- ACTACTATGGCGGGTGATAAAACGTGTAGCAAGCTGTAATCGACGGGAAGAGCATGCCCATCC-3′
S4	5′- ACGGTATTGGACCCTCGCATGACTCAACTGCCTGGTGATACGAGGATGGGCATGCTCTTCCCG-3′

Table S2. The mechanisms of different inhibitors for endocytosis pathways

**Inhibitors**	**Mechanisms**	**Concentrations**
Methyl-β-cyclodextrin	Cholesterol depletion/ Inhibiting caveolae-mediated endocytosis	100 μM
Dynasore	Inhibiting GTPase activity of dynamin1/2 and Drp 1	20 μM
Amiloride Hydrochloride	Inhibiting macropinocytosis	20 μM
Cytochalasin D	Inhibiting actin filament polymerization	20 μM
Nocodazole	Depolymerizing microtubules	20 μM

Supplementary Experimental Section

Preparation and characterization of tFNAs and OGP-tFNAs: Firstly, tFNAs were prepared via DNA self-assembling procedure (95℃ for 10min and 4℃ for 20min) in the Tris-maleate (TM) buffer (10 mM Tris-HCl, 50 mM $MgCl_2$, pH=8.0) according to the previous reports.[1] OGP were synthesized and purified (>95% purity) by Bankpeptide Biological Technology (Anhui, China). The OGP powder was dissolved in ddH_2O and then mixed with tFNAs with different ratios for 6 h at room temperature. Then the samples were ultrafiltered to remove free OGP (Millipore Amicon Ultra 30K device, 4000 rpm, 10 min for 3 times). Polyacrylamide gel electrophoresis (PAGE, 8%) was used to confirm the success production of tFNAs and OGP-tFNAs. The morphology of tFNAs/OGP-tFNAs was detected via atomic force microscope (AFM, SPM 9700, Shimadzu, Japan). Meanwhile, the size and zeta potential were detected via dynamic light scattering (DLS, Zetasizer Nano ZS, Malvern Instrument Ltd., Malvern, United Kingdom). The standard curve of OGP was measured using ultraviolet spectrophotometer at 214 nm and the absorption spectra of OGP-tFNAs with different OGP/tFNA ratios were also detected. Adsorption efficiency and loading capacity were also detected and presented d as follows:

$$\text{Adsorptiom efficiency} = \frac{\text{Initial mass of OGP} - \text{Residual mass of OGP}}{\text{Initial mass of OGP}} X\ 100\% \quad (1)$$

$$\text{Loading capacity} = \frac{\text{Initial molar number of OGP} - \text{Residual molar number of OGP}}{\text{Total molar number of tFNAs}} \quad (2)$$

In vitro drug release kinetics: 500 μL prepared OGP-tFNAs was sealed in the dialysis bag (30 kDa; Solarbio, Beijing, China), then the dialysis bag was immersed in 30 mL PBS at 37 ℃. The released OGP was detected using UV spectrophotometer and the total volume of the PBS was maintained unchanged during the experiment.

Stability test of OGP-tFNAs: OGP-tFNAs were mixed with 2%, 5% and 10% fetal bovine serum (FBS) for 0, 1, 3, 6, 9, 12, 24 and 48 h at 37 ℃. Finally, the samples were detected via agarose gel electrophoresis (1.8 g agarose/120 mL Tris-Borate-EDTA(TBE) buffer).

Cell culture and treatment: Bone marrow stromal OP9 cells were purchased from National Collection of Authenticated Cell Cultures (Shanghai, China). The cells were cultured in α-MEM with 1% penicillin-streptomycin and 20% FBS at 37 ° C in 5% CO_2. When the cell intensity approached 80%-90%, OP9 cells were recultivated in new α-MEM culture media. To study the potential effect of OGP-tFNAs on chemotherapeutics-induced hematopoietic damage and myelosuppression, OP9 cells were treated with tFNAs, OGP and OGP-tFNAs separately. After 12 h pretreatment, the cells were subsequently treated with 5-fluorouracil for another 24 h and then harvested for later detections.

Cellular uptake: To study the potential influence of OGP adsorption on the cellular internalization of tFNAs, and the influence of tFNAs as drug carriers on the cellular internalization of OGP, the tFNAs were labeled with Cy5 fluorescence and OGP was labeled with FITC fluorescence. After the cells were treated with tFNAs-Cy5 and OGP-FITC for 6 h and 12 h separately, the cells were rinsed with PBS for 3 times and harvested for flow cytometric analysis (Attune® NxT, Invitrogen, USA) and confocal fluorescence microscopy observation (FV3000, Olympus, Japan).

Cellular internalization mechanism of OGP-tFNAs: Five inhibitors (Methyl-β-cyclodextrin, MedChemExpress, USA; Dynasore, MedChemExpress, USA; Nocodazole, MedChemExpress, USA; Amiloride hydrochloride, Abcam, USA; Cytochalasin D, Abcam, USA) were used to inhibit the clathrin, caveolae,

macropinocytosis, actin microfilament, and microtubule. The OP9 cells were pretreated with five inhibitors separately in 2% FBS culture media for 1 h, then the culture media was replaced with new culture media simultaneously containing OGP-tFNAs-Cy5 and different inhibitors for another 6 h. After that, the samples were collected for flow cytometric analysis and confocal microscopy observation for fluorescence intensity detection.

Cell viability assay: Cell Counting Kit-8 (CCK-8) assay was applied to detect the cell viability after treatment with tFNAs, OGP and OGP-tFNAs with different OGP/tFNAs ratios for 24 h. Furthermore, CCK-8 assay was also used to select proper concentration of 5-FU (MedChemExpress, USA) from 6.25-100 μg/mL for the *in vitro* model of chemotherapeutics-induced OP9 cells injuries.

Cell cycle, senescence and apoptosis detection: Cell cycle and apoptosis were detected via flow cytometric analysis. OP9 cells were cultured in 6-well plates and followed by 12 h OGP/tFNAs/OGP-tFNAs pretreatment and following 24 h 5-FU treatment (25 μg/mL). The cell cycle was detected using cell cycle detection Kit (KeyGEN, Jiangsu, China). Generally, the cells were digested with 0.25% trypsin and fixed in 70% ethanol for overnight. After that, the samples were rinsed with PBS and incubated with RNase for 30 min in 37°C water-bath, then incubated with Propidium Iodide (PI) for 30min and detected via flow cytometry. The cell apoptosis was detected using Annexin V-FITC/PI apoptosis detection kit (KeyGEN, Jiangsu, China). After the cells were collected and resuspended with 500 μL binding buffer, 5 μL Annexin V-FITC and 5 μL PI were added into the binding buffer. After 5-15 min incubation, the samples were detected via flow cytometry. Furtherly, β-Galactosidase Staining Kit (Beyotime, Shanghai, China) was used to detect the senescent cells in different groups according to the standard protocol. After overnight incubation at 37°C in the staining solution, the samples were detected via microscopy observation.

Flow cytometric analysis for γ-H2AX expression in OP9 cells: OP9 cells were cultured in 6-well plate. After pretreatment with OGP, tFNAs and OGP-tFNAs for 12 h, the cells were treated with 25 μg/mL 5-FU for additional 24 h. Then the samples were collected and fixed in 70% ethanol at -20 °C for 1 h. After 3X washing with

staining buffer (2% FBS in PBS), then immunofluorescent staining was performed for later flow cytometry (PE anti-γ-H2AX, Biolegend, 1:20).

Western blotting: The total protein was extracted using Total Protein Extraction Kit (KeyGEN, Jiangsu, China) according to the standard protocol. After protein extraction and boiling denaturation, the proteins were separated using 10-15% SDS-PAGE and transferred to PVDF membranes. Then the samples were blocked with blocking buffer (QuickBlock™ Blocking Buffer, Beyotime Biotechnology, Shanghai, China) for 15min, and then incubated with related primary antibodies (anti-GAPDH, CST, 1:1000; anti-Bcl-2, CST, 1:000; anti-Bax, CST; anti-Caspase-3, CST, 1:1000; anti-Ki67, absin, 1:1000; anti-SCF, absin, 1:1000; anti-SDF-1, HuaBio, 1:500; anti-γ-H2AX, abcam, 1:5000) overnight at 4 ℃. At the second day, the samples were incubated with related secondary antibodies for 1h and detected using Gel & Blot Imaging system (ChemiDoc MP, Bio-Rad, USA).

Immunofluorescence staining: OP9 cells were cultured in 12-well plate and fixed with 4% paraformaldehydes after 12 h OGP/tFNAs/OGP-tFNAs pretreatment and another 24-h 5-FU treatment. The immunofluorescence staining procedure was performed according to the standard protocol. Briefly, the fixed samples were treated with 0.5% Triton-100 for 10 min and blocked with 5% goat serum (SL038, Solarbio) at 37 ℃ for 30 min. Then the samples were incubated with primary antibodies overnight at 4 ℃ and secondary antibodies for 1h at room temperature. After rinsed with PBS, the samples were stained with Acti-stain 488 phalloidin for 30 min and DAPI for 10min. Finally, the samples were detected using fluorescence confocal microscope (FV3000, Olympus, Japan).

Plasma pharmacokinetics: Plasma pharmacokinetics experiment was performed to investigate the in vivo metabolism of tFNAs and OGP-tFNAs. tFNAs-Cy5 and OGP-tFNAs-Cy5 were administrated via tail vein injection. The blood samples were collected via the tail tip of the mice before and 5 min to 24 h after injection. Finally, the blood samples were detected using the channel of Cy5 fluorescence of Gel & Blot Imaging system (ChemiDoc MP, Bio-Rad, USA). Pharmacokinetics was analyzed via the fluorescence intensity of the blood samples.

Myelosuppression induction and systematic drug delivery: The mice were divided into five groups: 1) Control group; 2) Saline + myelosuppression group; 3) tFNAs + myelosuppression group; 4) OGP + myelosuppression group; 5) OGP-tFNAs + myelosuppression group. For myelosuppression groups, the mice were continuously given 100 μl saline/OGP/tFNAs/OGP-tFNAs via tail intravenous injection days 1-9 and cyclophosphamide (150 mg/kg, MedChemExpress, USA) was administrated via intraperitoneal injection (i.p.) for 3 days (days 4-6) for myelosuppression induction. For normal group, the same volume of saline was delivered via tail intravenous injection at days 1-9 and PBS via intraperitoneal injection at days 4-6. At day 10, all the mice were sacrificed for later detections.

Biodistribution of tFNAs and OGP-tFNAs in bone marrow: Bone marrow smear was used to detect the biodistribution of tFNAs-Cy5 and OGP-tFNAs-Cy5 in the bone marrow after tail vein injection. At 3, 6, 12 and 24 h after injection, the femurs were collected for bone marrow smearing on the glass slides. Then the slices were stained with DAPI and detected via confocal microscopy observation.

Safety evaluation: The safety of tFNAs and OGP-tFNAs was evaluated after 2 and 5-times systematic delivery via tail vein injection. The major organs of the mice including heart, liver, spleen, lung and kidney were collected for later H&E staining for safety evaluation.

Histological detections: As main hematopoietic organs, femurs and sternums were collected and fixed in 4% paraformaldehyde. After decalcification for 3-4 weeks in EDTA decalcifying solution (Servicebio, Wuhan, China), the samples were dehydrated and embedded in paraffin. Then, the samples were coronally sectioned along the long axis of the femurs and sternums, then Hematoxylin-Eosin (H&E) staining was performed to observe the histological changes. Meanwhile, as major immune organ and metabolic organ, spleen and kidney were also collected for H&E staining. Besides H&E staining, femur samples were also sliced for immunohistochemical (IHC) staining to detect cell proliferation and hematopoiesis-related cell factors expression (anti-Ki67, absin, 1:200; anti-SCF, absin, 1:200; anti-SDF-1, HuaBio, 1:100). Furthermore, immunofluorescent staining was

also performed for femur samples to mark the Sca-1-positive and C-Kit-positive cells in the bone marrow (anti-Sca-1, Invitrogen, 1:250; anti-C-Kit, CST, 1:200).

References

[1] a) Y. Wang, Y. Li, S. Gao, X. Yu, Y. Chen, Y. Lin, Nano Letters **2022**, 22, 1759; b) M. Zhou, S. Gao, X. Zhang, T. Zhang, T. Zhang, T. Tian, S. Li, Y. Lin, X. Cai, Bioact Mater **2021**, 6, 1676.
